# Enzyme functionalized microgels enable precise regulation of dissolved oxygen and anaerobe culture

**DOI:** 10.1016/j.mtbio.2020.100092

**Published:** 2021-01-02

**Authors:** A.S. Jeevarathinam, F. Guo, T. Williams, J.A. Smolen, J.A. Hyde, M.J. McShane, P. de Figueiredo, D.L. Alge

**Affiliations:** a Department of Biomedical Engineering, Texas A&M University, College Station, TX 77843, USA; b Department of Microbial Pathogenesis & Immunology, Texas A&M Health Science Center, Riverside Parkway, Bryan, TX 77807, USA; c Department of Chemistry, Texas A&M University, College Station, TX 77843, USA; d Department of Materials Science and Engineering, Texas A&M University, College Station, TX 77843, USA; e Department of Veterinary Pathobiology, Texas A&M University, College Station, TX 77843, USA; f Norman Borlaug Center, Texas A&M University, College Station, TX 77843, USA

**Keywords:** Anaerobic culture, Gut microbe, Bioreactor, Hydrogel, Afterglow, Imaging

## Abstract

Anaerobes are a major constituent of the gut microbiome and profoundly influence the overall health of humans. However, the lack of a simple, cost-effective, and scalable system that mimics the anaerobic conditions of the human gut is hindering research on the gut microbiome and the development of therapeutics. Here, we address this gap by using glucose oxidase and catalase containing gelatin microparticles (GOx-CAT-GMPs) to precisely regulate dissolved oxygen concentration [O_2_] via GOx-mediated consumption of oxygen. Fluorescence images generated using conjugated polymer afterglow nanoparticles showed that [O_2_] can be tuned from 257.9 ​± ​6.2 to 0.0 ​± ​4.0 ​μM using GOx-CAT-GMPs. Moreover, when the obligate anaerobe *Bacteroides thetaiotaomicron* was inoculated in media containing GOx-CAT-GMPs, bacterial growth under ambient oxygen was comparable to control conditions in an anaerobic chamber (5.4 ​× ​10^5^ and 8.8 ​× ​10^5^ colony forming units mL^−1^, respectively). Finally, incorporating GOx-CAT-GMPs into a bioreactor that permitted continuous radial diffusion of oxygen and glucose generated a gut-mimetic [O_2_] gradient of 132.4 ​± ​2.6 ​μM in the outer ring of the reactor to 7.9 ​± ​1.7 ​μM at the core. Collectively, these results indicate that GOx-CAT-GMPs are highly effective oxygen-regulating materials. These materials can potentially be leveraged to advance gut microbiome research and fecal microbiota transplantation, particularly in low-resource settings.

## Highlights

• A large number of anaerobes are not studied owing to the limitations of current anaerobic culturing techniques.
• Here, we describe a novel but simple materials-based approach to culture anaerobes on a benchtop.
• The proposed approach eliminates the need for an anaerobic chamber or complex equipment.
• Oxygen-gradients generated in a bioreactor are verified by imaging oxygen-sensitive fluorescent nanoparticles.
• Suitability for anaerobic culture is demonstrated using the obligate anaerobe *Bacteroides thetaiotaomicron*.

## Graphical abstract

**Figure:**
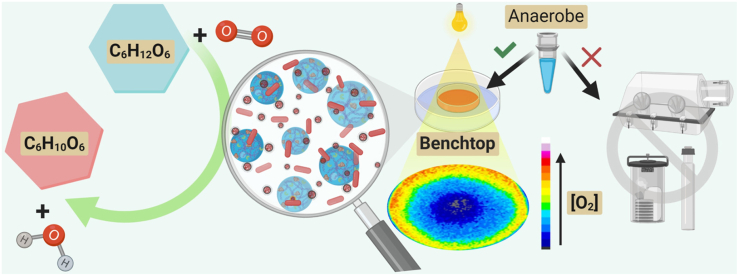

## 1 Introduction

Recent advancements in understanding the gut microbiome have strongly linked the gut flora to overall human health. A vast majority of gut microbiota found in the human colon are strict anaerobes, many of which remain unstudied because of a current lack of suitable anaerobic culture technologies [1,2]. For example, a recent metagenomics analysis on 11,850 human gut microbiome samples revealed 1952 novel uncultured bacterial candidates [3]. Difficulties with isolating and culturing these elusive anaerobes is currently limiting our understanding of their role in human health and disease as well as our ability to develop novel therapeutics from these microorganisms [4,5]. Therefore, innovative culture technologies that provide precise control over the *in vitro* microenvironment are needed to study the yet unexplored taxa of anaerobic bacteria [6]. The impact of novel anaerobic culture technologies would also extend beyond healthcare because of the enormous potential anaerobes hold for industrial and environmental biotechnology, for example in global nutrient cycles and the degradation of persistent compounds for soil remediation [5].

Several technologies have been developed to culture anaerobes in a laboratory environment, but they have notable limitations [6]. The Hungate's roll tube technique and its modern iterations involve the use of sealed tubes purged with anoxic gas to achieve anaerobic surface culturing [7,8]. Later, a technique commonly known as Gaspak, which involves the use of a vented anaerobic jar, initially developed by Brewer and Allgeier, came into use. Gaspak and its modifications leverage combustion to consume oxygen and generation or purging of hydrogen and carbon dioxide with a catalyst to remove residual oxygen [9]. The anaerobe chamber is an older but extensively used technique consisting of a flexible vinyl glove box based on the design of Arank et al. [10]. The anaerobic chamber uses a supply gas mixture containing hydrogen, carbon dioxide, and a noble gas to maintain an anaerobic atmosphere [11]. The addition of antioxidants to liquid media is another advancement that has enabled the cultivation of diverse strict anaerobes [12]. Nevertheless, the above techniques for handling and manipulating microbes are cumbersome or expensive. Moreover, they fail to replicate the gradient in oxygen concentration found in the human gut [13]. While recently developed gut-on-a-chip technology overcomes this challenge [14], this system involves either use of a dedicated anaerobic chamber or a degassed culture media coupled with precision pumps [[15], [16], [17], [18]]. Thus, there is still a critical need for simple, effective, scalable, and cost-effective culture methods, especially for use in low resource settings [19].

To advance the field, we aimed to create a simple bioreactor capable of generating an oxygen gradient that mimics the human gut. A key to realizing the above aim is to create a self-sustained biocatalyst to consume oxygen under precise control. Based on prior work on hypoxia-inducible hydrogels, we posited that oxygen-consuming biomaterials have the potential to address this need. For example, Park et al. reported that oxygen consumption during laccase-mediated dimerization of phenolic moieties conjugated to gelatin and dextran can be leveraged to induce hypoxia in 3D hydrogels [20,21]. However, the inherent link between oxygen consumption and functional group conversion during the gelation reaction limits the duration of oxygen depletion in these materials. Dawes et al. proposed an alternative system based on covalent immobilization of glucose oxidase (GOx), which consumes oxygen by the oxidation of glucose ​and demonstrated its suitability for 3D hypoxia culture of an acute myeloid leukemia cell line within poly(ethylene glycol) (PEG)–based hydrogels [22]. A major advantage of GOx for designing oxygen-consuming materials is that oxygen depletion can be sustained as long as the enzyme remains active and sufficient glucose is in the medium, even if oxygen is allowed back into the system. However, the potential of GOx for regulating [O_2_] spatially and generating gradients within a bioreactor and its utility for anaerobe culture have not been studied.

In this work, we describe the use of GOx and catalase (CAT)-loaded gelatin microparticles (GOx-CAT-GMPs) to precisely regulate [O_2_] and enable benchtop anaerobe culture. When GOx-CAT-GMPs were suspended in neutral aqueous medium containing glucose, the GOx enzyme oxidizes glucose to gluconolactone and depletes the dissolved oxygen in the medium, whereas CAT serves to prevent hydrogen peroxide accumulation. In contrast to an anaerobic chamber, which costs approximately $20,000, a batch of GOx-CAT-GMPs can be produced using readily available materials for less than $1 (estimates in USD). GOx-CAT-GMPs were synthesized via water-in-oil emulsion, and the effects of GOx loading were characterized using an established enzyme activity assay [23]. Subsequently, to test the efficacy of GOx-CAT-GMPs for regulating dissolved [O_2_], we performed experiments in which glucose was used as a limiting reagent and measured the dissolved [O_2_] with a commercial sensor. Poly[2-methoxy-5-(2-ethylhexyloxy)-1,4-phenylenevinylene] (MEH-PPV)-based afterglow nanoparticles (AGNPs) with oxygen-dependent afterglow [24] were also synthesized and incorporated to visualize and quantify GOx-CAT-GMP–mediated regulation of dissolved [O_2_] in a closed system (i.e. ​a sealed 96-well plate). The afterglow intensity measurements were correlated to [O_2_] as measured with precalibrated commercial oxygen sensor. To test the utility of these materials for microbial culture, the anaerobe *Bacteroides thetaiotaomicron* was added to GOx-CAT-GMPs dispersed in growth medium, and growth of the microbes outside of an anaerobic chamber was evaluated using a colony-forming unit (CFU) assay. Finally, the utility of GOx-CAT-GMPs to generate a gut-mimetic oxygen gradient in a bioreactor system that permitted continuous radial diffusion of oxygen was tested using afterglow imaging [24,25], where afterglow intensity measurements were used to spatially quantify dissolved [O_2_].

## 2 Materials and methods

### 2.1 Materials

Gelatin (from porcine skin gel strength 300; Cat No. G2500), glutaraldehyde (25% in water; Cat. No. G6257), microfiltered water (HPLC grade; Cat No. 34877), MEH-PPV (Mn ​= ​40,000-70000 ​KDa; Cat No. 541443), poly(ethylene glycol)-*block*-poly(propylene glycol)-*block*-poly(ethylene glycol) (PEG-*b*-PPG-*b*-PEG) (Mn ​= ​14,600; Cat No. 542342), brain heart infusion (BHI, Cat No. 53286), l-cysteine (Cat No. C7352), vitamin K (Cat No. 95271), and noble agar (Cat No. A5431) were purchased from Sigma Aldrich company, MO, USA and used without further purification. Light mineral oil (Cat No. S25439A) and dialysis tubing (Molecular weight cut-off ​12,000–14000 ​Da; Cat No. 21-152-14) were purchased from Thermo Fisher Scientific, MA, USA. Span 80 (Cat No. S0060) was purchased from TCI America, OR, USA. Bacto yeast extract (Cat no. 288620) was produced by BD Biosciences. Hematin (Cat no. 00812) was obtained from Chem-Impex International Inc., IL, USA.

### 2.2 Synthesis of GOx-CAT-GMPs, GOx-GMPs, and blank-GMPs

In a typical synthesis of GOx-CAT-GMPs, a solution of gelatin (12.5 ​wt %) in deionized water was prepared by dissolving gelatin (2.0 ​g) in microfiltered water (16.0 ​mL) at 60 ​°C. Then, the above solution (16 ​mL) was divided into four aliquots (each 4.0 ​mL) in separate centrifuge tubes and cooled to 42 ​°C in a water bath. Next, one aliquot (4.0 ​mL) of the above solution was quickly mixed with aqueous solution (0.5 ​mL) containing GOx (8.0 ​mg ​mL^−1^; 904.0 ​U ​mL^−1^) and CAT (0.12 ​mg ​mL^−1^_;_ 904.0 ​U ​mL^−1^). Then, aqueous glutaraldehyde solution (0.5 ​mL, 100 ​mM) maintained at 42 ​°C was added and mixed. The above mixture of gelatin, GOx, CAT, and glutaraldehyde was immediately added dropwise into light mineral oil (100 ​mL) and stirred at 800 ​rpm, 42 ​°C. The same process was repeated with the remaining three aliquots of gelatin solution to produce a water-in-oil emulsion. Stirring was continued at room temperature for 12 ​h. Finally, acetone (50.0 ​mL) was added to the above mixture, and the mixture was filtered under vacuum. The GOx-CAT-GMPs were washed with acetone (3 ​× ​30 ​mL), dried under vacuum, and stored at −20 ​°C.

To study the control of enzyme activity, GOx-GMPs, with only GOx (without CAT) was prepared using an identical procedure as was used for the synthesis of GOx-CAT-GMPs. However, an aqueous solution containing GOx (2.0–8.0 ​mg ​mL^−1^) was used in place of GOx and CAT solution used above (for preparation of GOx-CAT-GMPs). It should be noted that the GOx-GMPs lack the ability to contain the build-up of hydrogen peroxide. Hence, for all other studies except the enzyme activity assay, we used GOx-CAT-GMPs to eliminate the build-up of hydrogen peroxide. Similarly, blank GMPs were prepared under identical conditions by using pure deionized water instead of enzyme solution and used as negative controls.

### 2.3 Synthesis of AGNPs

AGNPs were prepared by a nanoprecipitation method. A solution of MEH-PPV (0.25 ​mg ​mL^−1^) was prepared by dissolving MEH-PPV (4.0 ​mg) in anhydrous tetrahydrofuran (16.0 ​mL) overnight with stirring at 300 ​rpm in dark conditions. Then, PEG-*b*-PPG-*b*-PEG (Mn ​= ​11,600; 320.0 ​mg) was dissolved in the above solution to achieve a final concentration of 0.25 ​mg ​mL^−1^ MEH-PPV and 20.0 ​mg ​mL^−1^ of PEG-*b*-PPG-*b*-PEG. Finally, the above solution (3.0 ​mL) was injected into microfiltered water within 30 ​s under continuous sonication at 150–200 ​W ​. After continuous sonication for 2 minutes, the tetrahydrofuran was evaporated by continuous bubbling of argon gas at 60 ​°C for 6 ​h. AGNPs were obtained as a bright red suspension and were concentrated to 3.0 ​mL by centrifuging at 3500 ​rpm in a 50 ​kDa cutoff centrifugal filter. The excess PEG-*b*-PPG-*b*-PEG was removed by washing the MEH-PPV NPs with Millipore water (6 ​× ​3.0 ​mL) using 50 ​kDa cutoff centrifugal filter.

### 2.4 Characterization

#### 2.4.1 GOx activity of enzyme cross-linked gelatin microparticles

The GOx activity of enzyme-loaded microparticles was characterized by the well-established *o*-dianisidine/horseradish peroxidase assay [23]. For this purpose, CAT-free gelatin microparticles loaded with different amounts of GOx were prepared, as described above. The use of CAT-free GMPs for activity assay is justified by the interference of CAT in the peroxidase-catalyzed oxidation of *o*-dianisidine by hydrogen peroxide.

#### 2.4.2 Absorption and fluorescence spectra

The fluorescence and absorption spectra of the AGNPs were recorded on a Tecan Infinite M200 Pro plate reader with AGNPs in water (200 ​μL, 125 ​μg ​mL^−1^). The concentration of the AGNPs suspensions was determined immediately after preparation using an Agilent Technologies Carey 300 UV–Vis spectrophotometer with a quartz cuvette with path length of 1.0 ​cm and sample volume of 3.0 ​mL. In brief, to determine the concentration, solutions of MEH-PPV in chloroform were prepared in the concentration range of 0–100 ​μg ​mL^−1^, and the absorbance at 495 ​nm was used to construct a calibration curve. Finally, the 100 ​μL AGNP as-prepared suspension was lyophilized and redissolved in chloroform to measure the absorbance.

#### 2.4.3 Afterglow activity of the AGNPs

All the samples were irradiated with 0.25 ​W ​cm^−2^ white light (400–900 ​nm). Excitation was done using a THORLABS OSL2 optic fiber illuminator equipped with an optic fiber bundle for different lengths of time as required in each experiment, and fluorescence images were acquired 30 ​s after switching off the light source.

The fluorescence afterglow images were acquired on a Bruker Xtreme 4 ​MP imaging cabinet equipped with a 4-megapixel cooled-CCD camera using the Molecular Imaging software suite (Bruker Inc. v. 7.2.0) at the Texas A&M institute for preclinical studies. For the single-exposure images, a 1-min ​exposure time was used with a f-stop of 1.1, a field of view of 72 ​mm, and 8 ​× ​8 pixel binning to enhance light sensitivity (final resolution was 92 pixels/inch). All imaging was performed at room temperature. The afterglow images were analyzed using the ImageJ Fiji [[26], [27], [28]]. Afterglow intensity was quantified in arbitrary units from pixel grayscale values (0–250) for precise measurement of oxygen. The afterglow images presented in the figures are in pseudo-color for visualization.

#### 2.4.4 Optimization of the irradiation time of samples

To optimize the time of irradiation for afterglow imaging, 200 ​μL of aqueous suspensions of MEH-PPV AGNPs with varying concentrations (150, 90, and 30 ​μg ​mL^−1^) were placed in a 96-well plate in triplicate. Then, the samples were irradiated from the top for different lengths of time (0–270 ​s with increments of 30 ​s) and imaged using the procedure described above.

#### 2.4.5 Limit of detection ​and optimal concentration of AGNPs

To determine the limit of detection (LoD) of the AGNPs, 200 ​μL of different concentrations of AGNPs (200-1 ​μg ​mL^−1^) were placed in a 96-well plate in triplicate. The sample sets were then separately irradiated with white light (0.25 ​W ​cm^−2^) from the top for 210 ​s and imaged as described above.

#### 2.4.6 Afterglow lifetime determination

For the afterglow lifetime determination, four identical samples with a volume of 100 ​μL were placed in four adjacent wells in a black clear bottom 96-well plate. Then, the samples were irradiated with 0.25 ​W ​cm^−2^ white light from the top and imaged as described above every 15 ​s for a total period of 10 ​min (0–600 ​s).

#### 2.4.7 Calibration of afterglow intensity and dissolved oxygen concentration

To correlate afterglow intensity to [O_2_], a suspension containing AGNPs (94 ​μL, 125 ​μg ​mL^−1^), GOx-CAT-GMPs (25 ​mg ​mL^−1^), blank GMPs (25 ​mg ​mL^−1^) (amounting to a total GMP concentration of 50 ​mg ​mL^−1^), and varying concentrations of glucose (7-0 ​mM) was used. The above suspensions were formed *in situ* in a black clear-bottom 96-well plate under a uniform layer of light mineral oil to limit diffusion of oxygen from the head space. Then, the samples were irradiated from the bottom with white light (0.25 ​W ​cm^−2^) for 210 ​s through a 0.5 ​cm thick cross-linked gelatin matrix (10% gelatin with 10 ​mM glutaraldehyde). This was done to mimic the setup of our bioreactor construct (see next section) and enable accurate correlation of the afterglow signal to [O_2_]. The total volume of each sample (96 ​μL) was calculated to correspond to the sample path length (3.1 ​mm) in the bioreactor construct. Finally, the [O_2_] in each of these samples were measured using a commercial electrochemical oxygen microsensor (UNISENSE OX-500) to draw a correlation between afterglow intensity and [O_2_]. Each measurement reported is an average of three independent repeats.

#### 2.4.8 Fabrication and afterglow imaging of the bioreactor construct

A simple bioreactor construct that was open to radial diffusion was fabricated to test the potential of the GOx-CAT-GMPs to produce an oxygen gradient. We used a 28.6 ​mm (diameter of cylinder) snake-skin dialysis tubing (regenerated cellulose) with a molecular weight cutoff value of 12–14 ​kDa to form a semipermeable core for the bioreactor system. First, a perforated (with 4.4 ​mm circular punches) inner support with aluminum foil (18 μ thick) and a black vinyl mask was placed inside a 2.0 ​cm long segment of dialysis tubing to keep it in hollow cylindrical form. Then, the dialysis tube with a perforated inner support was placed inside a petri dish (with diameter of 52.0 ​mm) containing an aqueous solution of gelatin (10.0 ​mL, 0.5 ​cm height; 10% w/w) and glutaraldehyde cross-linker (10 ​mM). After 10 ​min, the gelatin solution solidified into a transparent and leak-proof base around the dialysis tubing.

A total of six identical bioreactor constructs were fabricated and divided into control and experimental groups (with three reactors in each group). First, the reservoir (i.e. ​space between semipermeable core and the outer wall of the dish) in the reactors in both sets were filled with aqueous glucose solution (2.0 ​mM). The semipermeable core of the control group reactors was filled with suspension containing blank GMPs (2.0 ​mL, 50 ​mg ​mL^−1^) and of AGNPs (125 ​μg ​mL^−1^). The reactors in the experimental set were filled with suspension containing GOx-CAT-GMPs (2.0 ​mL, 25 ​mg ​mL^−1^), blank GMPs (25 ​mg ​mL^−1^), and AGNPs (125 ​μg ​mL^−1^). Finally, the reactor cores with GMPs and AGNPs in both experimental and control sets were topped with light mineral oil to limit oxygen diffusion from the top, spiked with 20 ​μL 200 ​mM glucose solution, and equilibrated for 5 ​min before imaging. For imaging, the bioreactor constructs were irradiated with white light from the bottom to avoid shadow effect of the semipermeable walls, and images were acquired as described above. The afterglow images of bioreactors were analyzed in image J to obtain the average pixel density (*Pixel density* ​= ​*Intensity/area of the zone*) and quantitatively map the oxygen distribution. For this purpose, the area of the bioreactor was divided into 10 different concentric circular zones. Finally, the pixel densities of each of the zones were plotted as a function of zone numbers. This method of zone-wise calculation of pixel densities eliminates the error that can possibly arise from pixel intensity obtained across a single line (diameter) drawn across the afterglow image of a reactor.

### 2.5 Anaerobe culturing

To demonstrate the utility of GOx-CAT-GMPs in culturing anaerobes, *Bacteroides thetaitaomicron* (*B. thetaitaomicron* ATCC 700349), which is a human commensal bacterium that cannot grow under aerobic conditions was used. A modified BHI ​agar and broth, which contains BHI (37.0 ​g ​L^−1^), yeast extract (2.5 ​g ​L^−1^), hematin (0.5 ​mg ​L^−1^), l-cysteine (0.5 ​mg ​L^−1^), vitamin K (0.2 ​mg ​L^−1^), glucose (2.0 ​g ​L^−1^), and with or without Noble agar (12 ​mg ​L^−1^) (agar/broth) ​was used to culture the *B. thetaitaomicron*. In brief, *B. thetaiotaomicron* (∼1.5 ​× ​10^3^ ​CFU) was inoculated in the wells of 96-well plates, which were divided into duplicates of five experimental groups (8 wells per group). The wells in group 1 contained only 150 ​μL of above media as prepared. Group 2 contained 50 ​mg ​mL^−1^ blank GMPs. Group 3 contained 25 ​mg ​mL^−1^ of blank GMPs and 25 ​mg ​mL^−1^ of GOx-CAT-GMPs. Group 4 contained 125 ​μg ​mL^−1^ AGNPs, 25 ​mg ​mL^−1^ of blank GMPs, and 25 ​mg ​mL^−1^ of GOx-CAT-GMPs. Finally, group 5 contained only 125 ​μg ​mL^−1^ AGNPs. The plates were subsequently incubated at 37 ​°C in an aerobic incubator (experimental set) or an anaerobic chamber (standard anaerobe culturing condition; control set) for 16 ​h. After the incubation, the bacteria were serially diluted and plated on BHI agar, and then, the plates were incubated in the anaerobic chamber for CFU ​counting.

## 3 Results and discussion

**Fig. 1:**
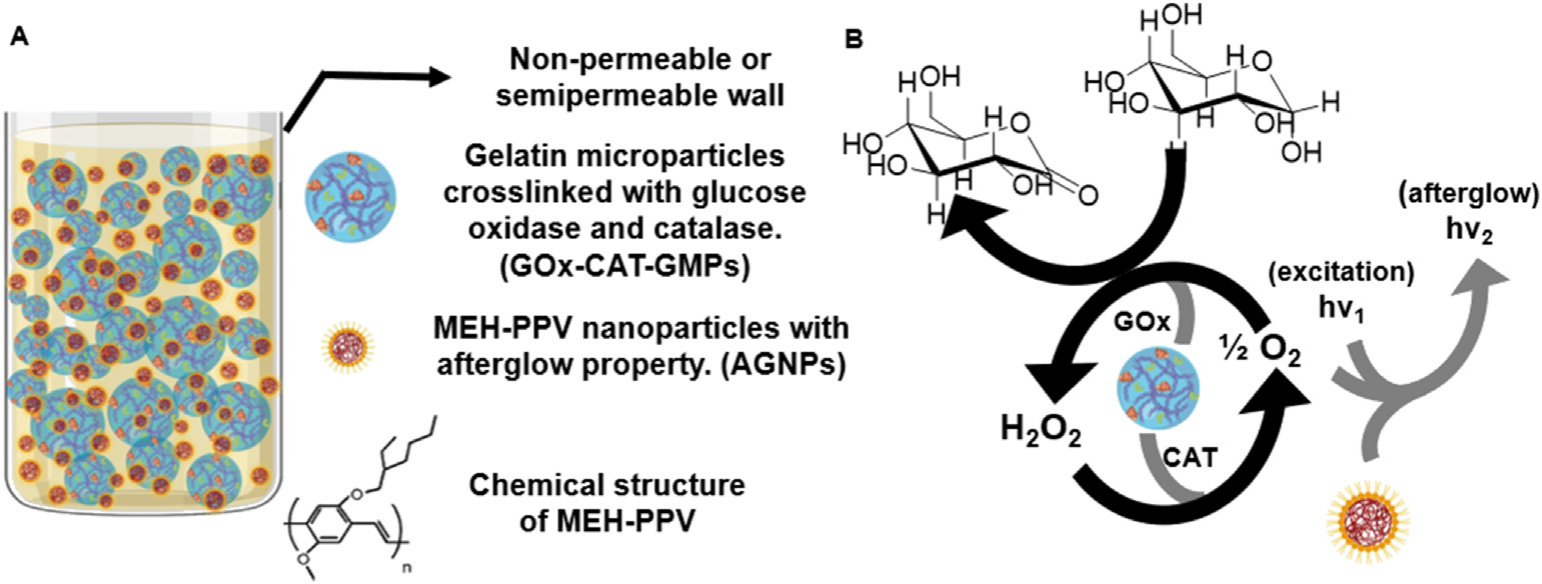
**Overview of materials used to regulate and quantify [O**_**2**_**]. (A)** Schematic diagram of the two-particle system. Gelatin microparticles (blue) were functionalized with GOx and CAT to deplete oxygen by oxidizing glucose in the culture medium. MEH-PPV nanoparticles (brown) are afterglow active and were used to quantify the oxygen level because afterglow intensity is proportional to [O_2_]. **(B)** Schematic illustration of oxygen consumption by GOx-CAT-GMPs and real-time oxygen imaging by AGNPs in the proposed dual-particle system. The stoichiometry shown in panel **(B)** of glucose oxidation by GOx and hydrogen peroxide decomposition by CAT results in eventual depletion of oxygen in the medium. GOx, glucose oxidase; MEH-PPV, Poly[2-methoxy-5-(2-ethylhexyloxy)-1,4-phenylenevinylene]; AGNPs, afterglow nanoparticles; CAT, catalase; GMPs, gelatin microparticles. (For interpretation of the references to color in this figure legend, the reader is referred to the Web version of this article.)

To address the need for a benchtop bioreactor system to culture anaerobe communities, we sought to circumvent the need for an anaerobic chamber by using oxygen-consuming hydrogel microparticles. Thus, we planned to bring about hypoxia by mixing the catalyst, GOx-CAT-GMPs in a medium that contained glucose and to image the spatial [O_2_] using AGNPs (Fig. 1A). As illustrated in the schematic representation (Fig. 1B), the stoichiometry of the GOx catalyzed oxidation of glucose [[29], [30], [31]], and CAT-catalyzed degradation of hydrogen peroxide [24] allowed complete depletion of dissolved oxygen in the presence of sufficient glucose in the medium. The AGNPs were used in the same medium to quantify and image the spatial oxygen distribution based on the oxygen-dependent afterglow intensity. From Fig. 1, it is clearly possible to control the concentration of glucose or the dissolved oxygen in a medium by using either one as a limiting reagent. Here, we demonstrate the control of [O_2_] in the culture media by using glucose as a limiting reagent in both closed and open systems.

### 3.1 Design and synthesis of oxygen-consuming hydrogel microparticles

**Fig. 2:**
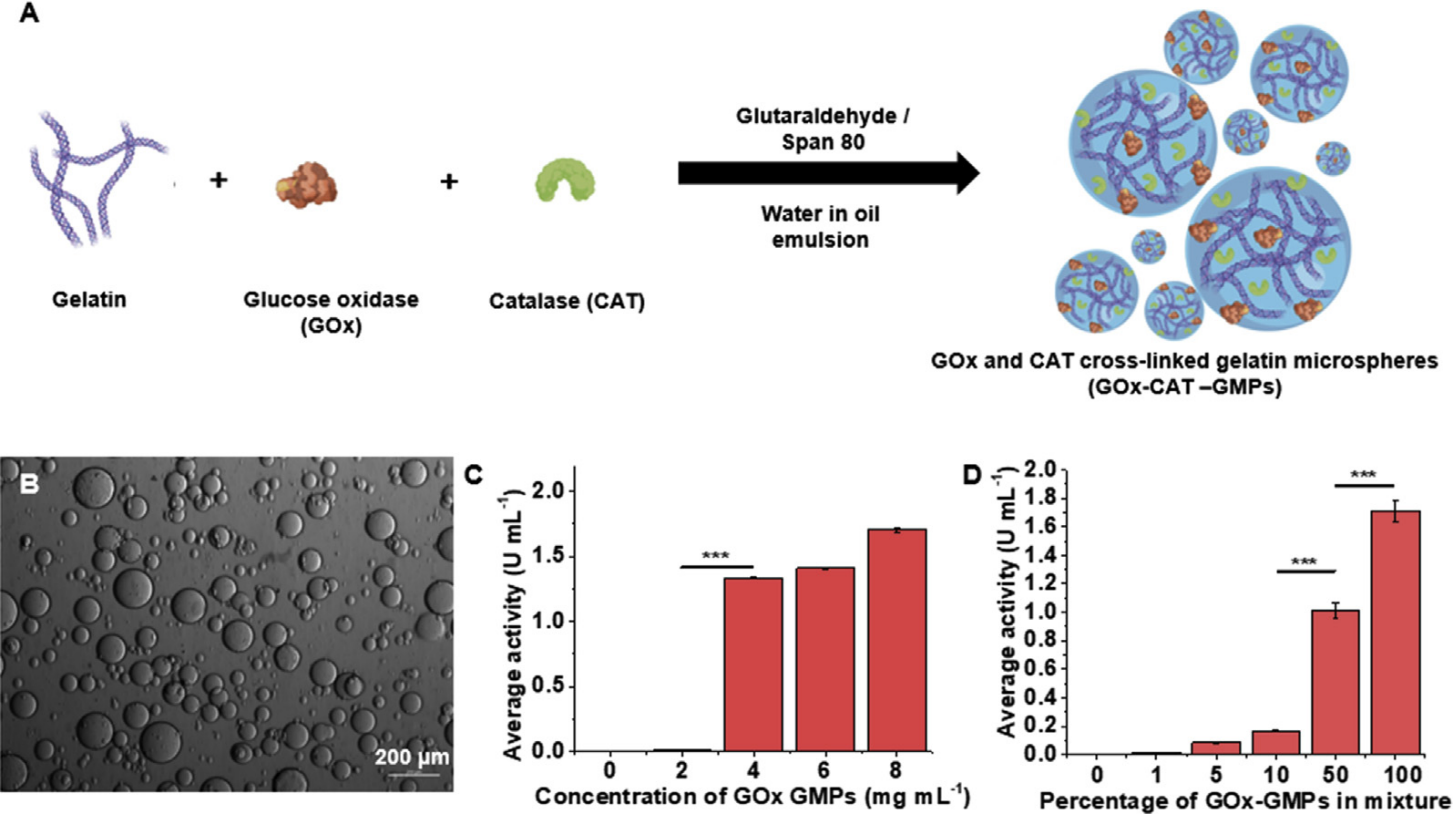
**Synthesis and characterization of gelatin microparticles containing glucose oxidase and catalase.** (**A**) Synthesis of glucose oxidase (GOx) and catalase (CAT) containing gelatin microparticles (GOx-CAT-GMPs). (**B**) Representative optical microscopy image of GOx-CAT-GMPs (5× magnification). (**C**) GOx activity of gelatin microparticles prepared with different feed concentrations of GOx (∗∗∗ indicates *p*<0.001; differences between the 4, 6, and 8 ​mg ​mL^−1^ groups were not statistically significant). (**D**) GOx activity of mixtures of GOx-containing GMPs (prepared with 8 ​mg ​mL^−1^ GOx) and blank gelatin microparticles. Note: GOx-GMPs were used for GOx activity determination instead of GOx-CAT-GMPs since catalase interferes with the enzyme activity assay (∗∗∗ indicates *p*<0.001; differences between the 1, 5, and 10% groups were not statistically significant).

First, we prepared enzyme-loaded gelatin (GOx or GOx-CAT) hydrogel microparticles using a water-in-oil emulsion method (Fig. 2A) because it is both simple and scalable. We dispersed a mixture of 10% w/w gelatin, 904 ​U ​mL^−1^ GOx (with or without 904 ​U ​mL^−1^ CAT), and 1 ​mM glutaraldehyde in deionized water into mineral oil containing 1.0% v/v Span 80 [32]. We selected gelatin as a support matrix to immobilize GOx because of its low cost, ease of handling, biocompatibility, and tunability as a hydrogel support matrix [[33], [34], [35]]. Further, hydrogels in general can be fine-tuned to achieve desired physicochemical properties [36]. We were able to repeatedly achieve high yields of GOx-CAT-GMPs (85–95%) over repeated syntheses (n ​= ​6) by mixing glutaraldehyde with gelatin and enzymes just before emulsification. The average diameter of the GOx-CAT-GMPs was found to be 65.1 ​± ​31.2 ​μm (average ​± ​standard deviation; n ​= ​194 particles, largest ​= ​182.2 ​μm, smallest size ​= ​4.9 ​μm). (Fig. 2B). This wide range of sizes is typical for hydrogel microparticle synthesis by the water-in-oil emulsion method [37,38].

For the purpose of assaying GOx activity, we omitted CAT and synthesized bioactive GOx-GMPs by only crosslinking GOx to gelatin using an identical procedure. We then used these GOx-GMPs to determine the best method to control the overall level of enzyme activity in the culture medium. We initially studied the GOx activity of the microparticles (GOx-GMPs) as a function of the GOx feed concentration. We prepared GOx-GMPs with GOx concentrations ranging from 226.0 to 904.0 ​U ​mL^−1^ (226.0, 452.0, 678.0, and 904.0) and achieved variable mean GOx activity of 0.0 ​± ​0.0 to 1.9 ​± ​0.2 ​U ​mL^−1^ (n ​= ​3) (Fig. 2C). However, while attempting to modulate the activity of GOx-GMPs by tuning the feed concentration of GOx, only low and high activities were obtained (difference in activities were statistically insignificant, *p*>0.05). Finer control of GOx activity from 0.0 ​± ​0.0 to 1.7 ​± ​0.1 ​U ​mL^−1^ was achieved by physically mixing different percentages (w/w) of GOx-GMPs (prepared with GOx feed of 904.0 ​U ​mL^−1^) with blank GMPs to reach an overall concentration of 10 ​mg ​mL^−1^ of gelatin particles in the dispersion (Fig. 2D). Thus, using mixtures of GOx-GMPs with blank GMPs was determined to be the better approach for controlling the overall amount of enzyme activity in aqueous dispersions of GOx-GMPs.

For subsequent experiments, gelatin microparticles containing GOx and CAT (GOx-CAT-GMPs) were used to prevent the build-up of hydrogen peroxide in the reaction medium and to improve longevity of enzyme immobilized microspheres [39,40]. This is important to preserve GOx activity over time and to mitigate detrimental effects of hydrogen peroxide accumulation on the growth of anaerobes. GOx-CAT-GMPs, GOx-GMPs, and blank GMPs had similar size ranges (Fig. S1).

### 3.2 Correlation of dissolved [O_2_] to afterglow intensity

#### 3.2.1 Synthesis of AGNPs

**Fig. 3:**
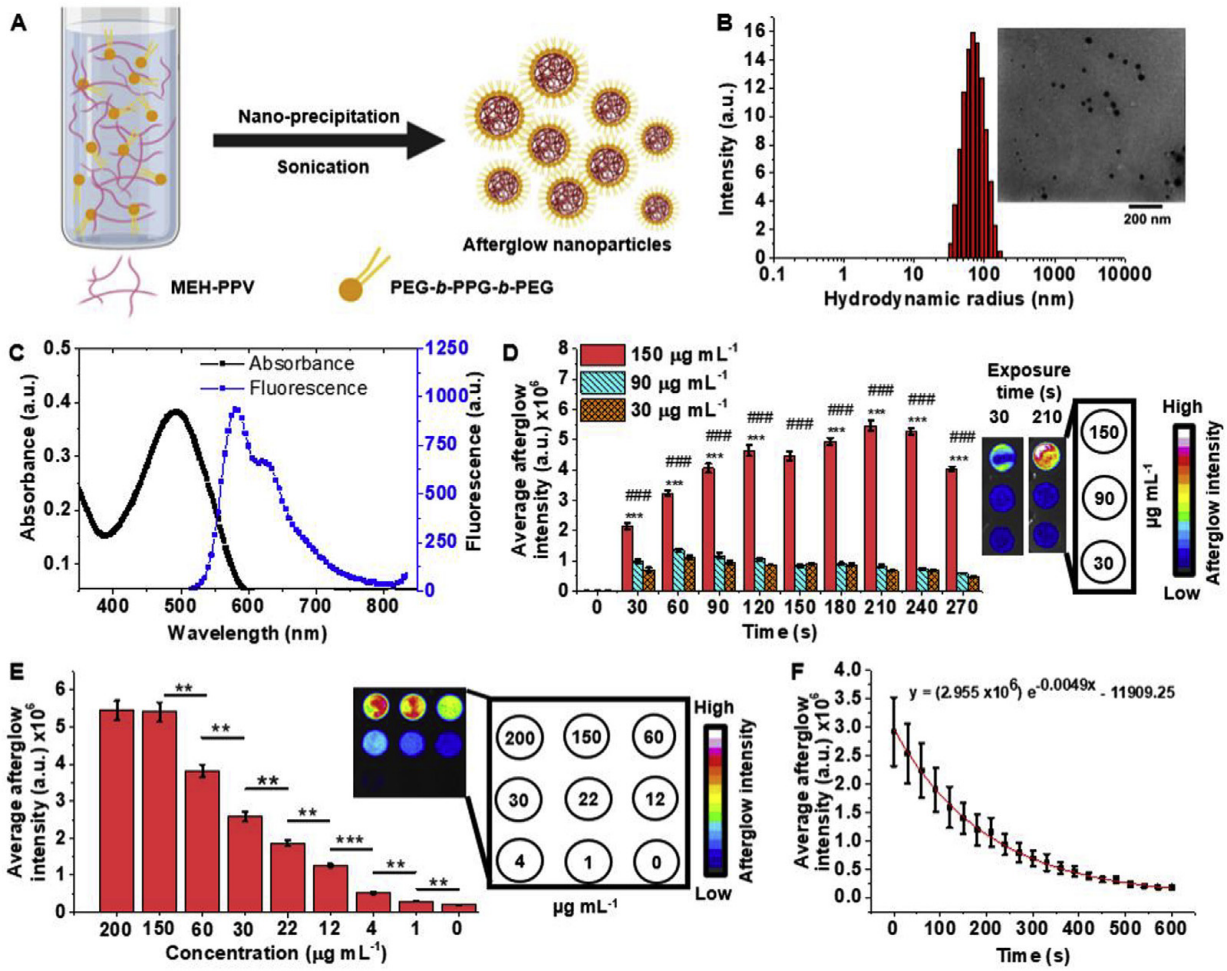
**Synthesis and characterization of MEH-PPV nanoparticles with oxygen-dependent fluorescence afterglow. (A)** Schematic illustration of the synthesis of AGNPs. **(B)** Distribution of hydrodynamic radii of prepared AGNPs ( ​mg ​100 μg mL^−1^) with inset showing a transmission electron micrograph ​of AGNPs (scale bar is 200 ​nm, drop cast from ​mg ​100 μg mL^−1^ suspensions). **(C)** Absorbance and fluorescence spectra of the AGNPs suspended in deionized water. **(D)** Evolution of afterglow intensity with increasing time of exposure to the excitation source (∗∗∗ indicates *p*<0.001 for comparisons between different time points for the 150 ​μg ​mL^−1^ suspension; ### indicates *p*<0.001 for comparisons between the 150 ​μg ​mL^−1^ and 90 and 30 ​μg ​mL^−1^ groups within same time point). **(E)** Change in afterglow intensity with different concentrations of AGNPs along with afterglow images of AGNPs at different concentrations (0–200 ​μg ​mL^−1^ as shown in the legend, and exposure time was 210 ​s; ∗∗ and ∗∗∗ indicate *p* ​< ​0.01 and *p*<0.001, respectively). **(F)** Lifetime trace of afterglow of AGNPs after excitation for 210 ​s, showing a half-life of 200 ​s. AGNPs, afterglow nanoparticles; MEH-PPV, poly[2-methoxy-5-(2-ethylhexyloxy)-1,4-phenylenevinylene]; PEG-*b*-PPG-*b*-PEG, poly(ethylene glycol)-*block*-poly(propylene glycol)-*block*-poly(ethylene glycol).

After developing our method to produce GOx-loaded hydrogel microparticles and characterizing their activity, it was necessary to include an oxygen imaging agent compatible with imaging the spatial distribution of [O_2_]. For this purpose, we synthesized MEH-PPV polymer-based AGNPs stabilized by PEG ​chains to serve as an oxygen imaging agent (Fig. 3A) [25]. These AGNPs exhibit a unique afterglow property that originates from a photochemical reaction between vinyl groups and oxygen to form an oxetane intermediate, which subsequently decomposes to emit photons [24,25]. The quantity of emitted photons is directly proportional to the amount of oxetane groups generated and, hence, to the [O_2_] around an AGNP [41]. The MEH-PPV–based AGNPs have been reported to have an afterglow lifetime spanning several seconds and are biocompatible for *in vivo* applications [24,41]. Inspired by these attributes of MEH-PPV, we specifically used commercially available MEH-PPV with number averaged molecular weight (M_n_) of 40–70 ​kDa and PEG-*b*-PPG-*b*-PEG with M_n_ ​= ​14.6 ​kDa to synthesize AGNPs through nanoprecipitation (Fig. 3A). The weighted average size of the AGNPs was 101.7 ​± ​21.6 ​nm according to TEM and 105.7 ​± ​13.4 ​nm according to DLS, which is larger compared with a previous report on MEH-PPV AGNPs (30–40 ​nm by DLS and 33.9 ​nm by TEM) (Fig. 3B) [24].

#### 3.2.2 Afterglow properties of AGNPs

To quantify [O_2_] in the medium using the prepared AGNPs, we studied their absorbance, fluorescence, afterglow lifetime, LoD, optimal concentration, and optimal time of exposure. Aqueous suspension of AGNPs showed an absorbance maximum at 495 ​nm and an emission maximum at 590 ​nm (Fig. 3C). Next, the optimal time of excitation of AGNPs was determined by exposing three different concentrations (30, 90, and 150 ​μg ​mL^−1^) of AGNPs to white light (0.25 ​W ​cm^−2^) for varying lengths of time (0–270 ​s with increments of 30 ​s). The afterglow intensity of the 30 and 90 ​μg ​mL^−1^, AGNP suspensions did not show a significant change for 90–210 ​s exposure (Fig. 3D, see Fig. S2). However, the 150 ​μg ​mL^−1^ suspension showed an increase in the afterglow intensity up to 210 ​s. Thus, we reasoned that 210 ​s was the optimal time of exposure within the concentration range of 0–150 ​μg ​mL^−1^ (Fig. 3D).

Using the optimized excitation time of 210 ​s, we studied the effect of AGNP concentration further and determined that the LoD of AGNPs in aqueous suspension was 4.0 ​μg ​mL^−1^ (Fig. 3E). Moreover, the afterglow intensity was directly proportional to concentration within the range of 0–150 ​μg ​mL^−1^. Above 150 ​μg ​mL^−1^, the difference in the afterglow intensity was insignificant (student's *t*-test; *p*>0.05). Based on these results, we chose 125 ​μg ​mL^−1^ AGNPs for imaging [O_2_] in reaction mixtures and in bioreactors. This concentration of AGNPs was chosen to avoid potential sensor saturation while also providing a high signal-to-noise ratio. The half-life of afterglow luminescence of the synthesized AGNPs was found to be 200 ​s (3.3 ​min) (Fig. 3F, see Fig. S3 for images). This long lifetime of the AGNPs is sufficient to allow a high signal-to-noise ratio because of elimination of background reflection.

We would like to point out that the MEH-PPV AGNPs synthesized in this work showed significant difference in size, LoD and afterglow half-life compared to previous reports. The differences in the photochemical and physical properties of our AGNPs compared with previous reports can be attributed to differences in size of AGNPs and the molecular weight of the surface amphiphilic groups used (PEG-*b*-PPG-*b*-PEG), both of which affect the surface availability of reactive vinylene groups. Based on the measured size (105.7 ​± ​13.4 ​nm), our synthesized AGNPs are estimated to have 3.1 times lower surface area compared with an equivalent volume of reported MEH-PPV nanoparticles (average diameter of 33.9 ​± ​4.3 ​nm) [24]. However, the observed percentage of variability in the hydrodynamic diameter was similar to reported value of 12.6%. Regardless, because the nature of reaction of AGNPs is a surface reaction, multiple readings are possible, and larger particles may mitigate potential loss of signal.

#### 3.2.3 Quantitation of GOx-CAT-GMP–mediated oxygen depletion

**Fig. 4:**
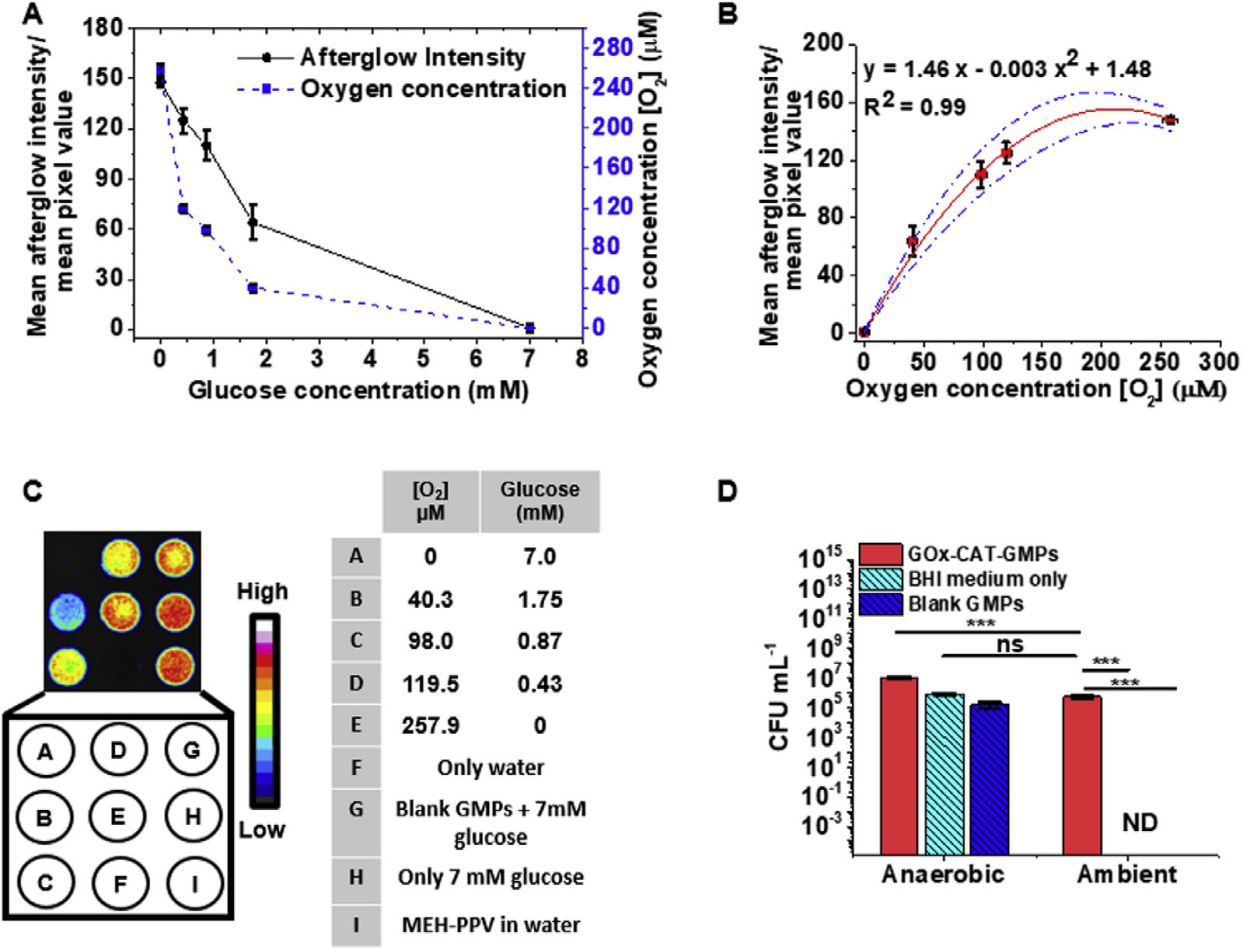
**Calibration of afterglow signal with [O**_**2**_**]. (A)** Afterglow response of the AGNPs to changes in glucose and [O_2_]. **(B)** Calibration of mean afterglow intensity (pixel density) with [O_2_]. A second degree polynomial fit with R^2^ ​= ​0.99 was obtained with statistically significant correlation (*p*<0.05 in ANOVA test) and was used in subsequent experiments to determine the spatial distribution of oxygen. **(C)** Representative afterglow images of the samples quantified in panels A and B, with a table identifying each sample (F–I are controls). The mean afterglow intensities represent an average of 4 replicates while [O_2_] values are averages of 10 replicates. **(D)** CFU counts of *Bacteroides thetaiotaomicron* in BHI growth media containing either 50 ​mg/mL of blank GMPs, 50 ​mg ​mL^−1^ of a 1:1 mixture of blank GMPs and GOx-CAT-GMPs, or only the BHI medium under ambient, aerobic conditions or inside an anaerobic chamber (∗∗∗ indicates *p*<0.001, ns indicates no statistically significant difference, ND indicates non-detectable, and n ​= ​3). GOx, glucose oxidase; MEH-PPV, Poly[2-methoxy-5-(2-ethylhexyloxy)-1,4-phenylenevinylene]; AGNPs, afterglow nanoparticles; CAT, catalase; GMPs, gelatin microparticles; BHI, brain heart infusion; CFU, colony forming unit.

Next, we sought to characterize oxygen depletion mediated by GOx-CAT-GMPs and to develop a mathematical relationship between [O_2_] and afterglow intensity of AGNPs present in the suspension. We used glucose as limiting reagent to control the concentration of dissolved oxygen in these suspensions. Accordingly, suspensions containing 50 ​mg ​mL^−1^ of GMPs (1:1 mixture of GOx-CAT-GMPs:blank-GMPs), 125 ​μg ​mL^−1^ AGNPs, and different concentrations of glucose were prepared and placed in a 96-well plate (94 ​μL each). When the concentration of glucose was varied from 0 to 7.00 ​mM (0, 0.43, 0.87, 1.75, and 7.00 ​mM), the [O_2_] was found to range from 257.9 ± 6.2 to 0.0 ± 4.0 (measured using a commercial Unisense OX500 oxygen sensor, see Fig. S4), whereas the afterglow intensity changed from 0 to 150 units (one-way ANOVA; *p*<0.05; See Fig. 4A–C) (Fig. S5 for more images). A plot of [O_2_] against the afterglow intensity was generated and fit to a polynomial curve (R^2^ ​= ​0.98) so that we could quantify spatial variations in [O_2_] in the bioreactor.

Care was taken during this correlation to keep the solution height (path length) uniform in each well at 94 ​μL (3.1 ​mm) (corresponding to Fig. 4A–C). All the wells were uniformly covered with 50 ​μL (∼1.5) mm of mineral oil to prevent oxygen diffusion from the headspace (see experimental section). The wells were also irradiated through the bottom of the plate through a 5.0 ​mm thick gelatin slab. This modification helped to ensure identical fluence of white light as used in the reactor in subsequent experiments. Irradiating from the bottom also helped to avoid the shadow effect of the walls (side-walls in 96-well plate). Finally, a high concentration of AGNPs (125 ​μg ​mL^−1^) was used to ensure abundant availability of contrast agent to measure the full range of [O_2_] and provide a high signal-to-noise ratio.

### 3.3 Anaerobe culture with GOx-CAT-GMPs

We next explored whether the hypoxic environment generated by GOx-CAT-GMPs can support culture of the obligate anaerobe outside of an anaerobic chamber. BHI growth media containing 50 ​mg ​mL^−1^ of a 1:1 mixture of GOx-CAT-GMPs and blank GMPs, unmodified BHI medium, or 50 ​mg ​mL^−1^ of blank GMPs were prepared. The obligate anaerobe *B. thetaiotaomicron* was inoculated in the above BHI broths in 96-well plates, and the wells were covered with mineral oil to prevent oxygen diffusion from the head space. The plates were then incubated at 37 ​°C either in an aerobic incubator (experimental set) or an anaerobic chamber (control set) for 16 ​h. Bacterial growth was then assessed by enumerating the CFU after the incubation. Importantly, the addition of GOx-CAT-GMPs not only supported *B. thetaiotaomicron* growth in the aerobic incubator but also achieved a comparable CFU count (5.4 ​× ​10^5^ ​CFU ​mL^−1^) relative to *B. thetaiotaomicron* grown in the unmodified BHI medium in an anaerobic chamber (8.8 ​× ​10^5^ ​CFU ​mL^−1^) (Fig. 4D). As expected, the BHI medium without GOx-CAT-GMPs did not support the growth of *B. thetaiotaomicron* when incubated in aerobic conditions. In addition, we noticed that GOx-CAT-GMPs improved the growth of *B. thetaiotaomicron* by 10-fold even in the anaerobic chamber (9.9 ​× ​10^6^ ​CFU ​mL^−1^) compared with unmodified BHI medium (8.8 ​× ​10^5^ ​CFU ​mL^−1^). We believe this significant increase in CFU is because of the consumption of the dissolved oxygen in the media. Interestingly, we also found that the addition of AGNPs to the modified BHI medium further improved the CFU count in the anaerobic chamber and under aerobic conditions (Fig. S6A). We attribute this effect to the oxygen scavenging property of AGNPs [24]. Finally, to assess the feasibility of anaerobic culture for longer periods of time, we evaluated GOx-CAT-GMP stability over time and found that there was no change in enzyme activity under these culture conditions after 7 days (Fig. S6B).

### 3.4 Generation of a radial oxygen gradient with GOx-CAT-GMPs

**Fig. 5:**
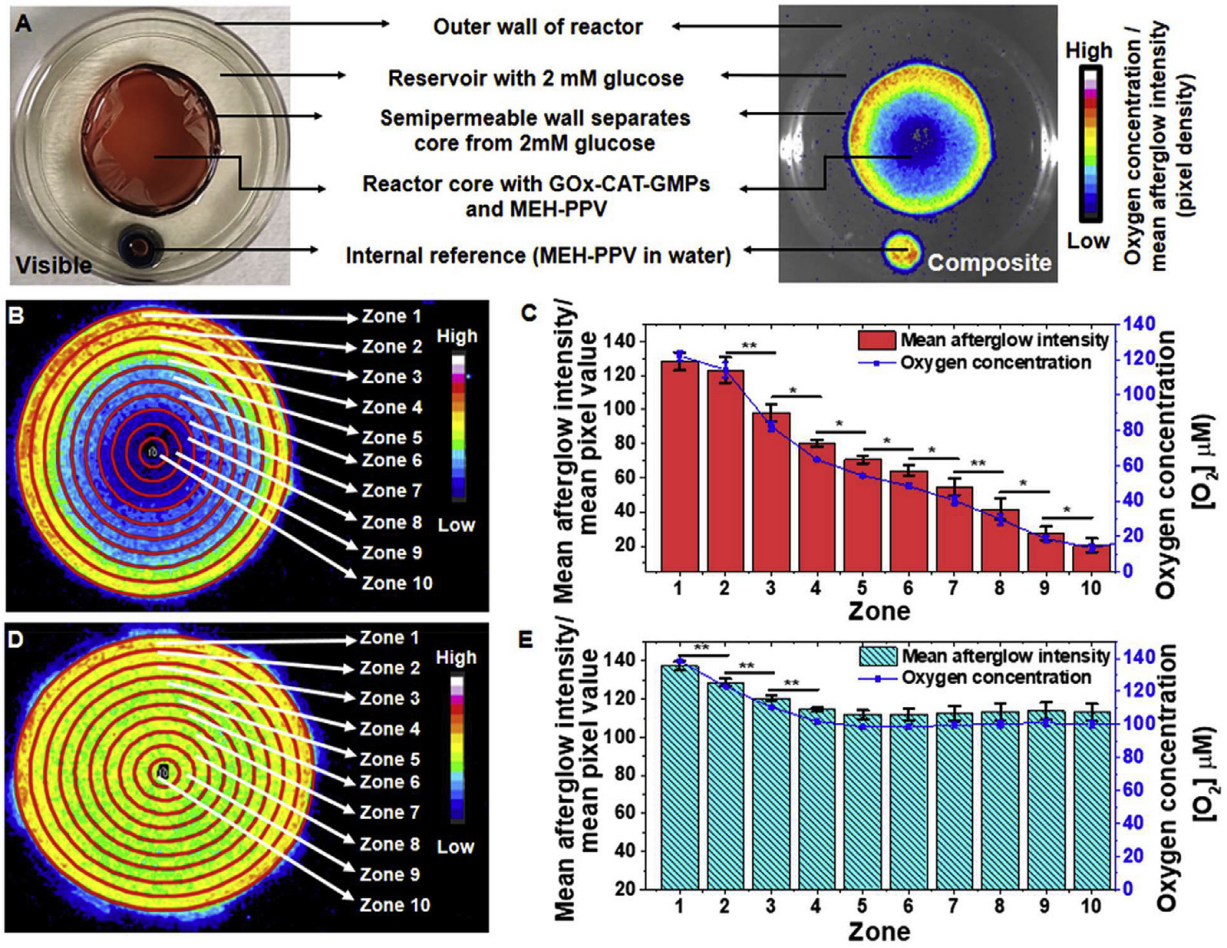
**Production of a radial oxygen gradient in a bioreactor with semipermeable walls.** (**A**) Visible and afterglow composite images of a representative bioreactor with an internal reference. **(B)** Representative afterglow image of a reactor in the experimental set with zones used to analyze the spatial distribution of oxygen across the cross-section. **(C)** Plot of the spatial distribution of afterglow intensity and corresponding [O_2_] across the different zones of the reactors from experimental set (n ​= ​3). **(D)** Representative afterglow image of a control reactor. **(E)** Plot of the spatial distribution of afterglow intensity and [O_2_] across the control reactors (n ​= ​3; ∗ indicates *p* ​< ​0.05 and ∗∗ indicates *p* ​< ​0.01). GOx, glucose oxidase; MEH-PPV, poly[2-methoxy-5-(2-ethylhexyloxy)-1,4-phenylenevinylene]; CAT, catalase; GMPs, gelatin microparticles.

In section 3.2.3, we correlated [O_2_] with afterglow intensity from AGNPs in a closed system (with no diffusion of oxygen and glucose). Next, we sought to use the GOx-CAT-GMPs to achieve a radial oxygen gradient within a simple bioreactor. In brief, the reactors were designed with a semipermeable wall surrounded by a reservoir with a non-permeable outer wall, and GOx-CAT-GMPs were loaded into the core (Fig. 5A). Thus, this design allowed glucose and oxygen from the reservoir to continuously diffuse radially through the semipermeable wall of the reactor core. Six such identical bioreactors were fabricated (see Fig. S7) and divided into experimental and control sets, with three reactor constructs in each set.

The semipermeable core of bioreactors (Fig. 5A) in the experimental set were filled with 2.0 ​mL (3.1 ​mm height) of a reaction mixture containing 50 ​mg ​mL^−1^ of 1:1 GOx-CAT-GMPs: blank-GMPs, 125 ​μg ​mL^−1^ of AGNPs, and 2 ​mM glucose. The core of the control sets contained 50 ​mg ​mL^−1^ of blank GMPs (enzyme free), 125 ​μg ​mL^−1^ of AGNPs, and 2 ​mM glucose. The reservoir (see Fig. 5A) of all the reactors (control and experimental sets) were filled with 2.0 ​mM glucose solution (3.1 ​mm height).

GOx-CAT-GMPs present in the core of experimental set reactors were expected to continuously deplete the oxygen that radially diffused into the core, resulting in a radial oxygen gradient. In contrast, no such reaction was possible in the control set lacking GOx-CAT-GMPs, and no gradient was expected. Afterglow images of the bioreactors, which were acquired using the same protocol used for calibration of afterglow intensity with oxygen level, confirmed these trends. In the experimental set, there was an obvious oxygen depletion in the center of the reactor core due to GOx-CAT-GMPs consuming the radially diffused oxygen (see Fig. S7). Using the polynomial fit established earlier in this work, the average oxygen gradient for the experimental set was found to range from 132.4 ​± ​2.6 ​μM at the outer periphery (Zone 1) to 7.9 ​± ​1.7 ​μM at the center (Zone 10) (Fig. 5B and C), corresponding to a 1676% difference ([O_2_] gradient of 16.7) in [O_2_] (n ​= ​3 reactors). In contrast, no significant [O_2_] gradient was observed in the control set with blank GMPs (Fig. 5D). Rather, the control set showed only a small variation from 164.3 ​± ​0.6 ​μM at the periphery to 99.6.0 ​± ​2.0 ​μM at the center of the reactor core (n ​= ​3 reactors) (Fig. 5E). Based on these results, GOx-CAT-GMPs are capable of generating varying degrees of hypoxia under continuous diffusion of glucose and oxygen into the reactor. Moreover, the conditions produced mimic those of the human gut with an oxygen partial pressure in the range of <40 ​mm Hg [13]. Based on recent work on gut-on-a-chip devices that possess similar spatial oxygen gradients [42], we expect that this bioreactor will enable the culture of diverse communities of microbes such as those found in the human gut.

## 4 Conclusion

Here, we have successfully demonstrated that oxygen consuming hydrogel microparticles can be used to regulate dissolved [O_2_], which we validated using AGNPs. While we used GOx containing gelatin hydrogel microparticles in this study, the same approach could be extended to other oxidase enzymes and polymer matrices if desired. Importantly, GOx-CAT-GMPs were highly effective at depleting oxygen in a closed system, in which they enabled the culture of an obligate anaerobe outside of an anaerobic chamber. Moreover, in an open bioreactor system that permitted radial diffusion through a semipermeable membrane to replenish depleted glucose and oxygen, they resulted in a gut-mimetic radial oxygen gradient with the lowest [O_2_] at the center of the reactor core. The inherent advantages of this bioreactor include robustness in control, ease of handling, and the ability to culture anaerobes without complicated instrumentation, which could be particularly valuable for use in low resource settings. Future studies will investigate the tunability of the oxygen gradient, for example by varying the concentration of GOx-CAT-GMPs in the reactor, and the utility of our materials and bioreactor for culturing diverse communities of microbes such as those of the gut microbiome.

## Data availability

The raw/processed data required to reproduce these findings cannot be shared at this time due to technical or time limitations but are available upon request.

## CRediT authorship contribution statement

**A.S. Jeevarathinam:** Investigation, Methodology, Visualization, Formal analysis, Writing - original draft. **F. Guo:** Investigation, Writing - review & editing. **T. Williams:** Investigation, Writing - review & editing. **J.A. Smolen:** Methodology, Resources. **J.A. Hyde:** Resources, Writing - review & editing. **M.J. McShane:** Resources, Funding acquisition, Writing - review & editing. **P. de Figueiredo:** Conceptualization, Supervision, Project administration, Funding acquisition, Writing - review & editing. **D.L. Alge:** Conceptualization, Supervision, Project administration, Funding acquisition, Writing - review & editing.

## Declaration of Competing Interest

The authors declare that they have no known competing financial interests or personal relationships that could have appeared to influence the work reported in this paper.
