# Supplementary material for: Enzyme functionalized microgels enable precise regulation of dissolved oxygen and anaerobe culture
Source: Mater Today Bio. 2021 Jan 2;9:100092. doi: 10.1016/j.mtbio.2020.100092 (PMC7856461; doi:10.1016/j.mtbio.2020.100092)
Supplement: Multimedia component 1 [file mmc1.pdf]

Supporting Information

Enzyme functionalized microgels enable precise regulation of dissolved oxygen and anaerobe culture

*Ananthakrishnan Soundaram Jeevarathinam, Fengguang Guo, Tyrell Williams, Justin A. Smolen, Jenny A. Hyde, Michael J. McShane, Paul de Figueiredo, and Daniel L. Alge**

Characterization of the stability of enzyme activity in GOx-CAT-GMPs.

To measure GOX-CAT-GMP activity over an extended period of time GOX-CAT-GMPs were suspended in bacterial culture media (identical to the media described in section 2.5 of the main text) at a concentration of 40 mg mL $^{-1}$ and their initial activity was measured using the procedure described in section 2.4.1 in the main text (3 aliquots of 1 mg mL^{-1}; n=3). Next, the entire batch was divided into two parts and filled in separate semipermeable tubes with molecular weight cutoff of 13-14 KDa. The two similar semipermeable dialysis tubes containing 2.0 mL of the GOx-CAT-GMPs suspension in culture media were sealed and placed in two separate larger containers with 30 mL of the same culture media. Then, the entire set up was incubated at 37 °C. After 7 days, 100 μL aliquots of the particle suspension were withdrawn and diluted to 1 mg mL $^{-1}$ (3 aliquots per batch; n=6). The enzyme activity in the diluted particle aliquots was then measured via the exact procedure described in section 2.4.1 in the main text. The results are presented as the average ± standard deviation in **Figure S6B**.

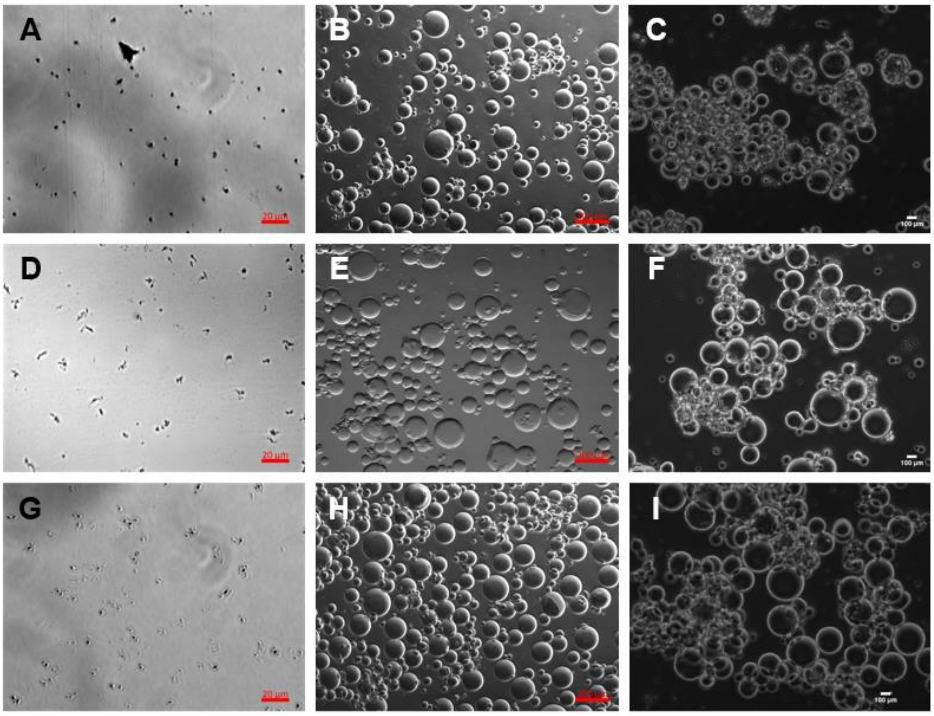

Figure S1. Micrographs of gelatin microparticles (GMPs) used in the study. (A) shows the dry glucose oxidase and catalase crosslinked gelatin microparticles (Gox-CAT-GMPs) dispersed in mineral oil. Panels (B) and (C) are GOx-CAT-GMPs dispersed in water with obvious swelling at 5x and 10x magnifications, respectively. Panel (D) shows dry catalase-free gelatin microparticles (Gox-GMPs) suspended in mineral oil. (E) and (F) presents the Gox-GMPs suspended in water with significant swelling at 5x and 10x magnifications, respectively. Panel (G) presents the dry blank-GMPs (enzyme-free) suspended in mineral oil and (H) and (I) are micrographs of blank-GMPs suspended in water at 5x and 10x magnifications, respectively. The micrographs clearly show that GOx-CAT-GMPs, Gox-GMPs, and blank-GMPs have similar size distribution.

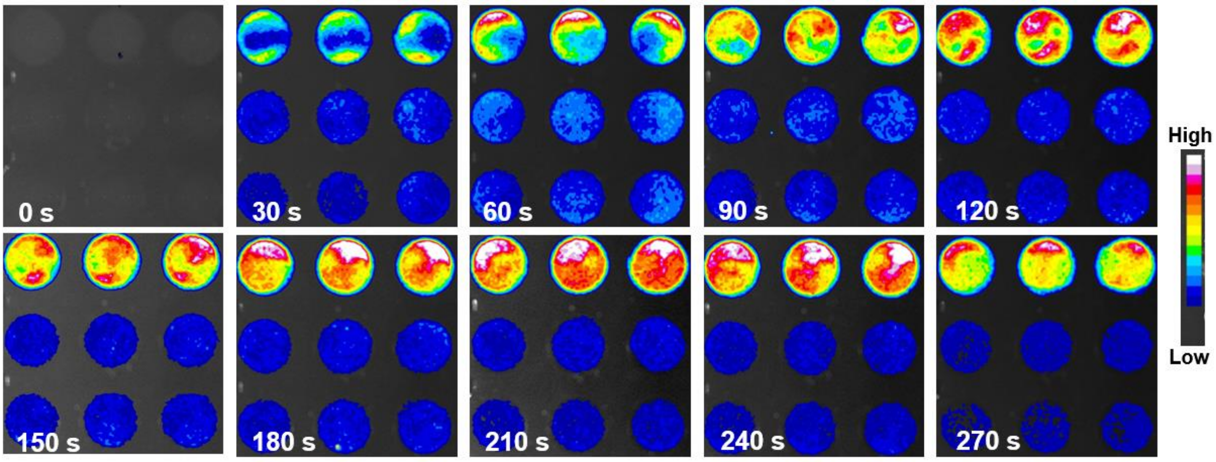

Figure S2. Afterglow images for time optimization. The evolution of afterglow intensity at different time of excitation is clearly visible from the above images. Each panel corresponds to different time of exposure to white light (0.25 W cm^{-2}) excitation. The first row in each panel is aqueous suspension 150 µg mL^{-1} of MEH-PPV afterglow nanoparticles AGNPs, the second row in each panel corresponds to 90 µg mL^{-1}, and the third row in each panel is 30 µg mL^{-1} AGNPs. Each column in every panel represents three repetitions (n=3). These panels correspond to Figure 2D.

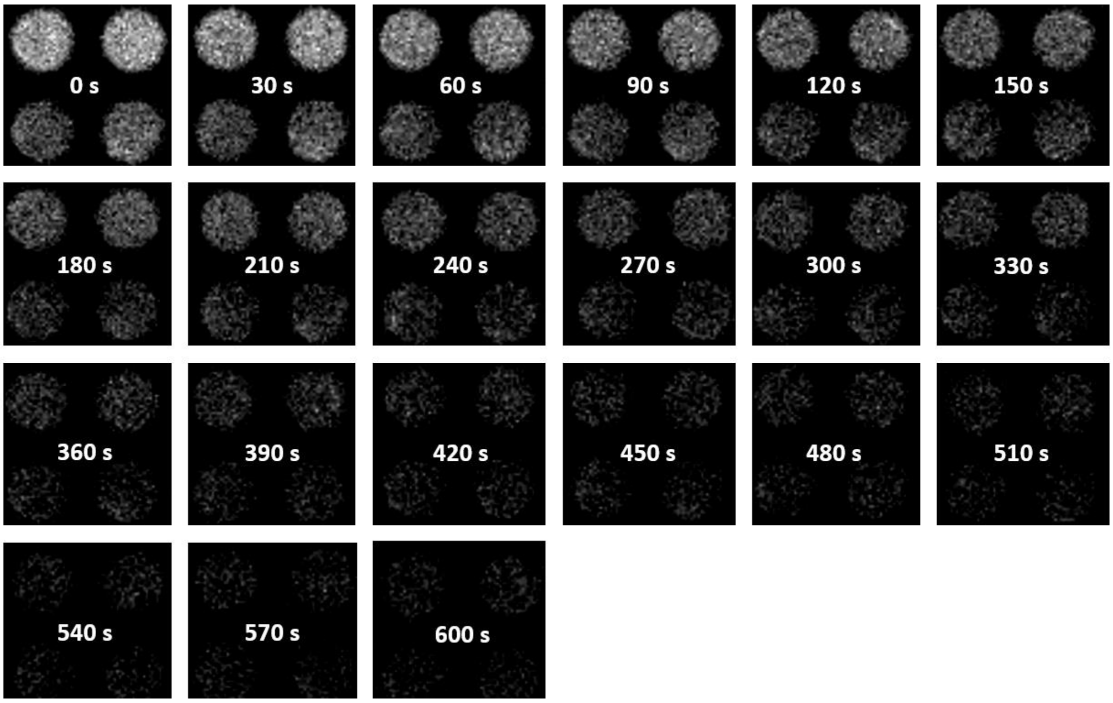

Figure S3. Images time decay of afterglow from AGNPs. The afterglow images corresponding to each time points (0-600 s) from the time dependent decay of afterglow signal from AGNPs.

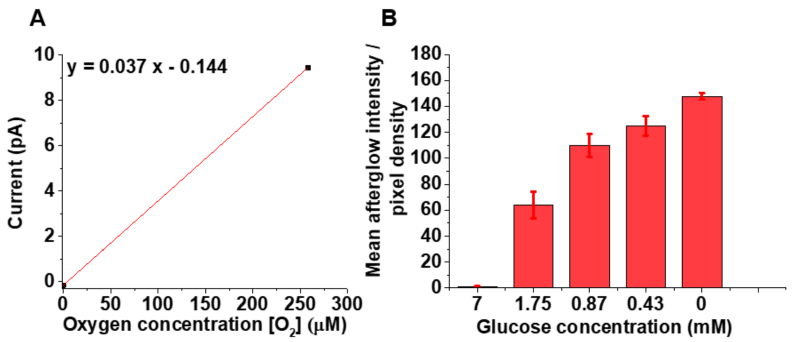

Figure S4. Calibration of electrochemical sensor (UNISENSE OX500) and effect of glucose on afterglow. Panel (A) shows the calibration curve of the UNISENSE OX500 oxygen sensor with equation used to correlate the afterglow intensity with oxygen concentration. Panel (B) shows the glucose dependent afterglow of AGNPs.

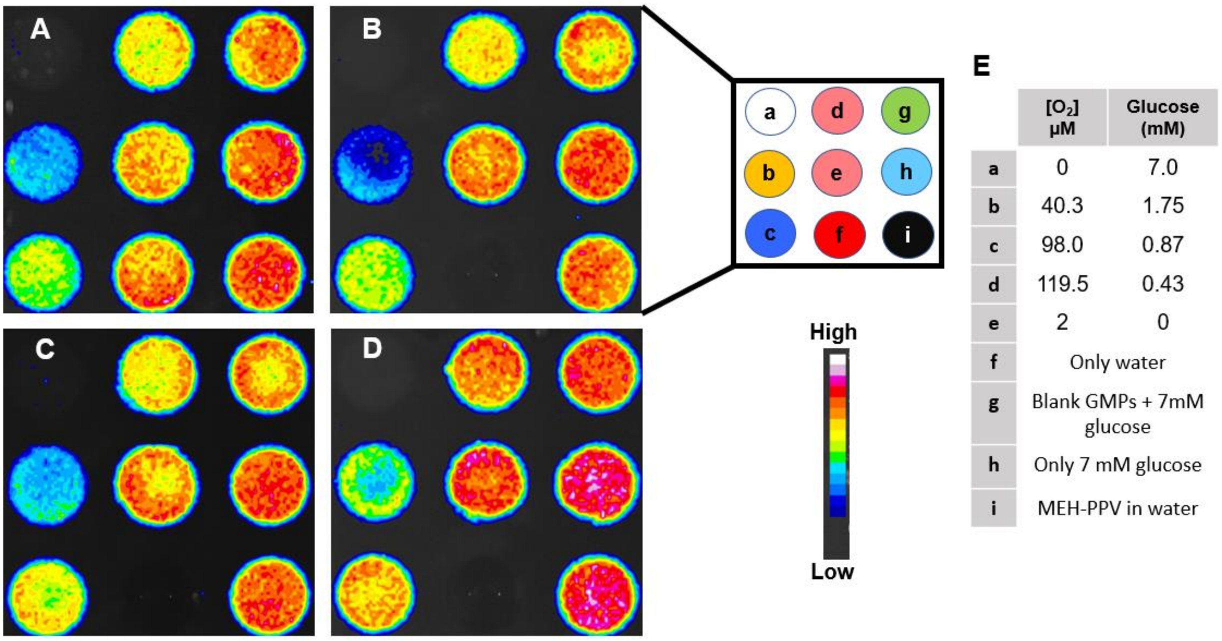

Figure S5. Correlation of afterglow with oxygen concentration. Panels (A-D) are Images of reactions mixtures containing 1:1 mixture of GOx-CAT-GMPs:blank-GMPs (total concentration of 50 mg mL^{-1}, 125 µg mL^{-1} AGNPs and corresponding glucose concentration as indicated in legend presented in panel (E). In panel A the wells e and f are same (50 mg mL^{-1}, 125 µg mL^{-1} AGNPs and 0 mM glucose) in contrast to other panels. However, in panel (A) the negative control (water) was placed in adjacent row (out of the frame presented)

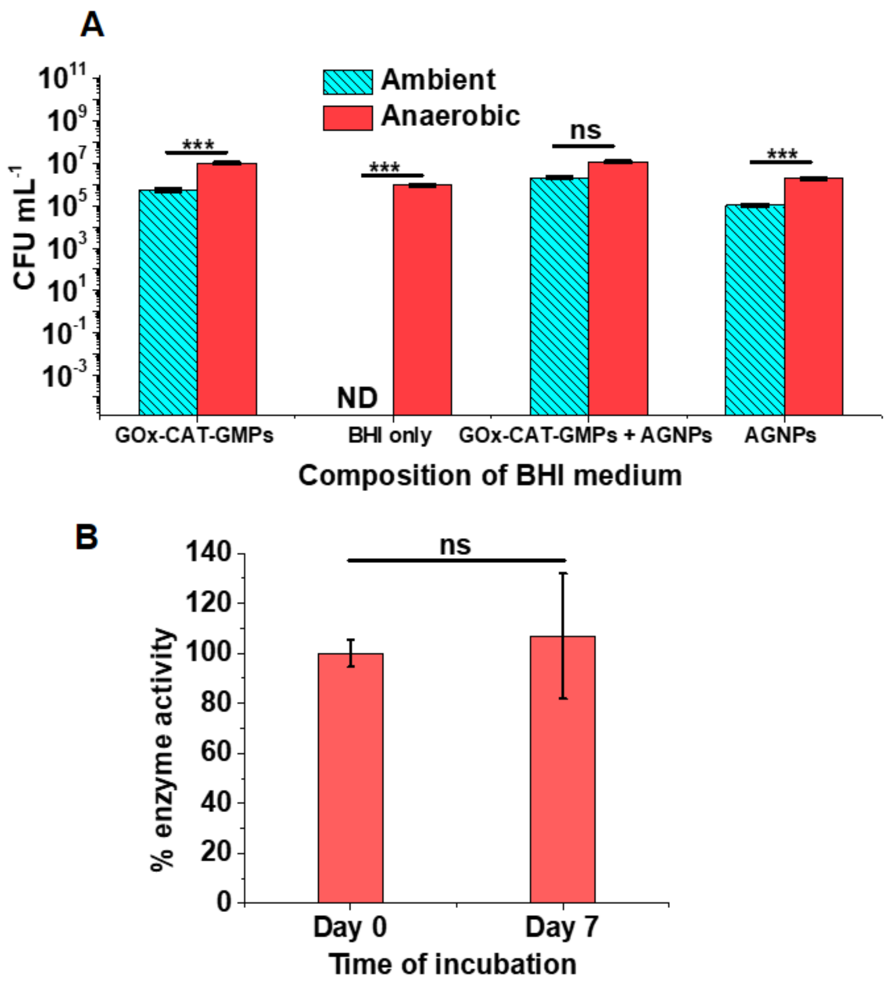

Figure S6. (A) Additional data on *Bacteroides thetaioataomicron.* CFU count for medium containing AGNPs show that the growth is enhanced in presence of AGNPs in the BHI media. Gox-CAT-GMPs are 25 mg mL^{-1} where mentioned and AGNPs are 125 µg mL^{-1} where mentioned. **(B)** The enzyme activity of GOx-CAT-GMPs incubated in the culture medium (containing 2 g L^{-1} glucose) for 7 days at 37 °C and the enzyme activity were assayed on day 0 (n=3) and at day 7 (n=6). No considerable change in enzyme activity was observed ($p>>0.05$).

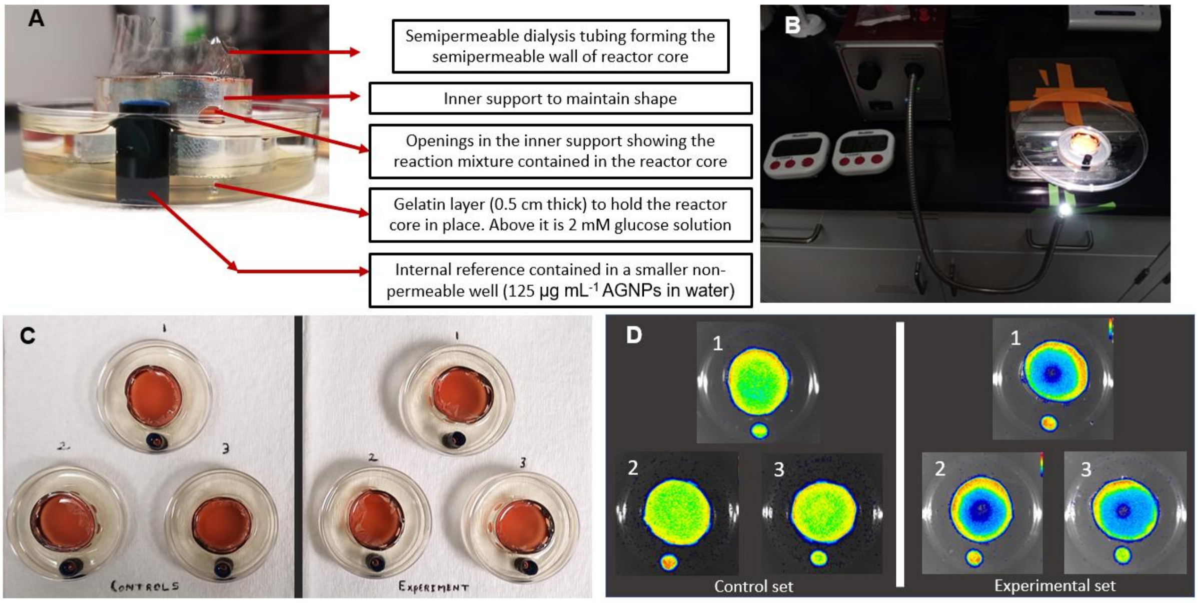

Figure S7. More details on Bioreactor construct and experiment set up. Panel (A) is the side view of a bioreactor construct shown in Figure 5A with different parts labeled. Panel (B) is the experimental set up used to excite reaction mixture in bioreactors. Panel (C) shows the image of all the bioreactor constructs fabricated and used in this study. The reaction mixture in the reactor core are red in color due to the presence of 125 µg mL^{-1} AGNPs. Panel (D) is the afterglow images of each of the bioreactors imaged under identical conditions.
